# Analysis of the Prognostic Value and Gene Expression Mechanism of *SHOX2* in Lung Adenocarcinoma

**DOI:** 10.3389/fmolb.2021.688274

**Published:** 2021-06-28

**Authors:** Nanhong Li, Yu Zeng, Min Tai, Biyun Lin, Di Zhu, Yi Luo, Xinle Ren, Xiaoying Zhu, Lanlan Li, Hongrong Wu, Jian Huang

**Affiliations:** ^1^ Institute of Nephrology, Affiliated Hospital of Guangdong Medical University, Zhanjiang, China; ^2^ The Center of Pathological Diagnosis and Research, Affiliated Hospital of Guangdong Medical University, Zhanjiang, China; ^3^ Department of Respiration, The Second Affiliated Hospital of Guangdong Medical University, Zhanjiang, China; ^4^ Department of Pathology, Guangdong Medical University, Zhanjiang, China

**Keywords:** *SHOX2*, methylation, expression, clinical parameter, LUAD, bioinformatic platform analysis

## Abstract

**Background:** Detection of *SHOX2* methylation has been used to assist in the early diagnosis of lung cancer in many hospitals as *SHOX2* may be important in the tumorigenesis of lung cancer. However, there are few studies on the mRNA expression, methylation, and molecular mechanism of *SHOX2* in lung cancer. We aimed to explore the role of *SHOX2* in lung adenocarcinoma (LUAD).

**Methods:** First, we examined the differential expression of *SHOX2* mRNA and methylation in cancerous and normal tissues using databases. Second, we analyzed the relationship between *SHOX2* expression and common clinical parameters in LUAD patients. Third, we further explored the methylated level and its specific location of *SHOX2* and the mainly factors of *SHOX2* gene expression. Finally, we screened the correlatively expressed genes to analyze the pathways from the Gene Ontology and Kyoto Encyclopedia of Genes and Genomes using DAVID.

**Results:** We found that the mRNA expression of *SHOX2* was higher in multiple cancers, including LUAD and lung squamous cell carcinoma (LUSC), than in normal tissues. Among LUAD patients, *SHOX2* expression was higher in patients of middle–young age, with smoking history, in advanced stages, and with nodal distant metastasis. In addition, our results showed that patients with high expression of *SHOX2* are prone to recurrence, poor differentiation, and poor prognosis. Thus, we identified that *SHOX2* might be an oncogene for LUAD progression. The main factor influencing the high expression of *SHOX2* mRNA may be DNA methylation, followed by copy number variation (CNV), but not by gene mutations in LUAD. Unexpectedly, we found that *SHOX2* undergoes hypomethylation in the gene body instead of hypermethylation in the promoter. Additionally, SHOX2 has cross talk in the PI3K–Akt signaling pathway and ECM–receptor interaction.

**Conclusion:** *SHOX2* is highly expressed in most cancers. *SHOX2* gene expression might be mainly regulated by methylation of its gene body in LUAD, and its high expression or hypomethylation indicates poor differentiation and poor prognosis. SHOX2 could be involved in PI3K–Akt and other important cancer-related signaling pathways to promote tumorigenesis.

## Introduction

Much progress has been made in the research field of lung cancer occurrence and progression. However, epidemiological investigations have shown that lung cancer has higher morbidity and mortality than other malignant cancers in China (Chen et al., 2016; Wu et al., 2019). The alarming statistical results of lung cancer remain mainly attributed to the late detection, late diagnosis, late treatment, and unsatisfactory therapeutic effect. In lung cancer, driver gene alterations are critical in the whole process of tumorigenesis, recurrence, and metastasis. More representative driver genes as biomarkers for accurate early diagnosis and prognostic evaluation and therapeutic targeted molecules could improve treatment efficiency and increase mortality. Therefore, the identification of these molecular biomarkers for lung cancer is of great clinical significance.

Short stature homeobox 2 (*SHOX2*) is highly orthologous to murine Shox2 and human *SHOX* (Blaschke et al., 1998; De Baere et al., 1998; Marchini et al., 2016; Hu et al., 2018). According to current studies on *Shox2*, *SHOX*, and SHOX2, *SHOX2* is considered to play a critical role in idiopathic short stature and various types of cardiac arrhythmias (Mortensen et al., 2012; Marchini et al., 2016). However, in recent decades, many researchers have found that *SHOX2* also plays an important role in multiple cancers, including lung cancer (Schneider et al., 2011; Hong et al., 2014; Sun et al., 2019). Interestingly, methylation detection of *SHOX2* in sputum, blood, alveolar lavage fluid, and tissue of patients has been used to screen early lung cancer patients (Zhao et al., 2015; Weiss et al., 2017; Zhang et al., 2017; Shi et al., 2020). It is worth noting that there is little research on the potential prognostic influence and molecular mechanism of *SHOX2* in lung cancer, especially in non-small-cell lung cancer (NSCLC) [mainly lung adenocarcinoma (LUAD) and lung squamous cell carcinoma (LUSC)]. The clinical significance of *SHOX2* research as an early diagnostic gene is beyond doubt.

In our research, data from The Cancer Genome Atlas (TCGA) were mined using several online analysis tools to evaluate the *SHOX2* expression profile in NSCLC. The main factors of the *SHOX2* gene expression mechanism, including mutation, copy number, and methylation, were then investigated. The relationship between *SHOX2* expression and the clinical characteristics of LUAD was determined using publicly accessible databases. Finally, potential co-expressed genes and functional networks were analyzed to provide direction for further investigation into the mechanism of how *SHOX2* works in lung cancer.

## Methods

### Oncomine Database Analysis

Oncomine is a large oncogene chip database that can be used to analyze and compare gene expression between tumors and normal tissues, as well as gene mutation profiles and their correlation with clinical characteristics (Rhodes et al., 2007). We used the Oncomine database to determine differences in the mRNA expression of the *SHOX2* gene between tumors and normal tissues in various cancers. The results of this analysis are addressed with a *p*-value of 0.05, a fold change of 2, all gene rankings, and mRNA data type.

### GEPIA

Gene expression profiling interactive analysis (GEPIA) is a web tool that provides key interactive analysis and customization capabilities, including tumor/normal differential expression profilometry, profile mapping, pathological staging, patient survival analysis, similar gene assay analysis, and dimensionality reduction analysis (Tang et al., 2017). We used GEPIA to address the differential expression of *SHOX2* in all common cancers. We applied the following cut-off criteria: using the ANOVA method, |log2 FC| > 1, *p*-value < 0.01, and log2(TPM +1) for log-scale, matching TCGA and GTEx normal data, and adding all cancer tissue names.

### GEO Microarray Analysis

The Gene Expression Omnibus (GEO; https://www.ncbi.nlm.nih.gov/geo/) is a database that stores chips, second-generation sequencing, and other high-throughput sequencing data worldwide (Edgar et al., 2002). An mRNA expression dataset (GSE33532) for early stage NSCLC was downloaded from the GEO database. GSE33532 stored mRNA expression information of 80 NSCLC tissue samples and 20 matched distant-normal samples from 20 patients. Microarray expression profiling was normalized by log2 transformation before analysis. These 20 patients were included in the validation dataset. The histological types of these NSCLC samples included adenocarcinoma, squamous cell carcinomas, and mixed carcinomas. Finally, we used GraphPad Prism 7 software to analyze and plot the collected data using unpaired *t*-test statistical methods. The *p*-value < 0.05 was considered statistically significant.

### SurvivalMeth Analysis

SurvivalMeth (http://bio-bigdata.hrbmu.edu.cn/survivalmeth/) is a web server that is freely available to address the prognostic information of cancer-associated methylation, based on TCGA, CCLE, and GEO (Zhang et al., 2020). We used SurvivalMeth to investigate the effect of *SHOX2* DNA methylation–related functional elements on protein expression and LUAD prognosis. We applied the following cut-off criteria: the chosen disease of lung adenocarcinoma, the chosen experimental platform of 450 K (Illumine Infinium HumanMethylation450 BeadChip), the chosen gene symbol of *SHOX2*, transcript-related elements including “1st exon, 3′UTR, 5′UTR, gene body, TSS1500, and TSS200,” CpG island–related elements including “Island, N_Shelf, N_Shore, S_Shelf, and S_Shore,” unrestricted repeat element–related element and CTCF-binding region, statistical methods using *t*-test, a threshold value of 0.05, and median group strategy.

### UCSC Xena Analysis

The University of California Santa Cruz Cancer Genomics Browser (UCSC Xena) (http://xena.ucsc.edu/) is an online database of genomic, transcriptomic, and clinical and phenotypic data (Kent et al., 2002; Zweig et al., 2008; Navarro Gonzalez et al., 2021). To investigate the dominant factors influencing *SHOX2* expression in LUAD, UCSC Xena was used to analyze the relationship between the methylation level, copy number, mutation, Kaplan–Meier survival analysis, and mRNA expression of *SHOX2* in TCGA LUAD samples. We set the following conditions: sample types including solid tissue normal and primary tumor, gene expression using RNAseq-IlluminaHiSeq, DNA methylation using Methylation 450K, copy number using (gene-level)-gistic2, and somatic mutation using (SNP and INDEL)-MC3 public version. Additionally, we used this tool to analyze *SHOX2* expression patterns in different pathological types.

### UALCAN Analysis

UALCAN (http://ualcan.path.uab.edu) is an online database that provides free visual figures of gene expression, survival analysis, correlation analysis, and gene DNA promoter region methylation data, grouped by clinicopathological features between normal and tumor tissues, based on TCGA data (Chandrashekar et al., 2017). To explore the relationship between *SHOX2* and the clinicopathological features of NSCLC patients, the expression and methylation levels of *SHOX2* in NSCLC and adjacent normal tissues were identified using UALCAN.

### MethSurv Analysis

MethSurv (https://biit.cs.ut.ee/methsurv/) used methylation group data from the “Cancer Genome Map” to perform survival analysis on the methylation patterns of CpG to achieve a preliminary assessment of tumor biomarkers based on methylation (Modhukur et al., 2018). We used MethSurv to further explore the relationship between *SHOX2* methylation and several clinical characterizations in a heat map.

### cBioPortal Database Analysis

The cBioPortal database (v3.5.4) (http://www.cbioportal.org) was used to perform the cancer genomic analysis (Gao et al., 2013; Jing et al., 2019). We chose two datasets, “Lung Adenocarcinoma (TCGA, Firehose Legacy)” and “Lung Adenocarcinoma (TCGA, Nature 2014),” to analyze the co-expressed *SHOX2* genes. We then set the following parameters: select genomic profiles [mutations, putative copy number alterations from GISTIC, and mRNA expression z-scores relative to diploid samples (RNA Seq V2 RSEM)], select patient/case set (all samples), and enter genes (user-defined list, *SHOX2*). Subsequently, we entered the co-expression interface and acquired the genes correlated with *SHOX2* in satisfactory samples from these datasets. These data were downloaded, and the next operating steps were followed.

### Using the Venn Diagram Tool

We selected the first 300 *SHOX2* correlated genes from the previous steps with a higher Spearman correlation coefficient and *p* < 0.05. According to the applied cBioPortal database, one gene list was named “TCGA, Firehose Legacy” and the other gene list was named “TCGA, Nature 2014.” We input the two lists into the upload lists. Then, we acquired the Venn diagram (http://bioinformatics.psb.ugent.be/webtools/Venn/) of *SHOX2* co-expressed related genes.

### Functional and Pathway Enrichment Analysis

The Database for Annotation, Visualization and Integrated Discovery (DAVID) (version 6.8; https://david.ncifcrf.gov/) is a biological information database that integrates biological data and analysis tools to provide systematic and comprehensive annotated biological function information for large-scale gene or protein lists to help users extract biological information from them (Huang da et al., 2009). We used DAVID to perform Gene Ontology (GO) analysis and Kyoto Encyclopedia of Genes and Genomes (KEGG) analysis of the selected common *SHOX2* co-expressed related genes screened by the two datasets. GO enrichment analysis consisted of cellular component (CC), biological process (BP), and molecular function (MF) analyses. Following these steps, we uploaded these genes, selected the identifier “OFFICIAL_GENE_SYMBOL,” selected “Homo spaiens,” selected “Gene List,” submitted the list, and finally obtained functional annotation charts. We then acquired the GO functional enrichment and KEGG pathway enrichment results. Calculated *via* Fisher’s exact test, a *p*-value < 0.05 and a count >2 were considered statistically significant.

## Results

### *SHOX2* mRNA Expression Profile in Multiple Cancers

**FIGURE 1:**
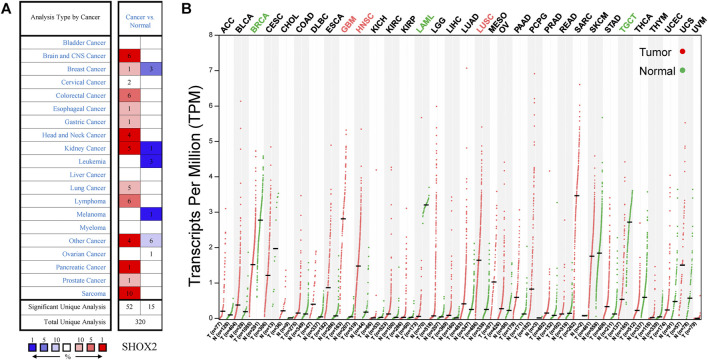
*SHOX2* mRNA expression in multiple cancers. **(A)** The number of datasets with *SHOX2* mRNA differential expression between tumor tissues and normal tissues. The left column (red) represents overexpression, and the right column (blue) represents downexpression. This visualized graphic is available from Oncomine, and the threshold parameters were set as follows: *p*-value = 0.05, fold change = 2, all gene rankings, and mRNA data type. **(B)** *SHOX2* gene expression profile in various tumor samples and normal samples by GEPIA. *SHOX2*: short stature homeobox 2; GEPIA: gene expression profiling interactive analysis.

To determine the expression level of *SHOX2* in various types of tumors, we examined the differential expression of *SHOX2* between tumor tissue and paired normal tissue using Oncomine and GEPIA online analysis tools. The Oncomine database has a total of 320 unique analyses for *SHOX2*. There are 52 significant unique analyses among them that showed *SHOX2* upexpression, while 15 analyses showed *SHOX2* downexpression (Figure 1A). *SHOX2* expression was higher in most cancers, including sarcoma, brain and CNS cancer, head and neck squamous carcinoma (HNSC), and lung cancer. Meanwhile, it was downregulated in breast cancer, leukemia, and many other cancers (Figure 1A). For further verification, we used the GEPIA web tool to compare the differential expression of *SHOX2* between cancerous and normal tissues. As shown in Figure 1B, higher *SHOX2* expression was observed in glioblastoma (GBM), HNSC, and LUSC. In addition, lower *SHOX2* expression was observed in breast invasive carcinoma (BRCA), acute myeloid leukemia (LAML), and testicular germ cell tumors (TGCTs). In summary, the results from the two databases were consistent, supporting the evidence that *SHOX2* should be an oncogene.

### Analysis of *SHOX2* Expression in LUAD/LUSC and Normal Tissues

**FIGURE 2:**
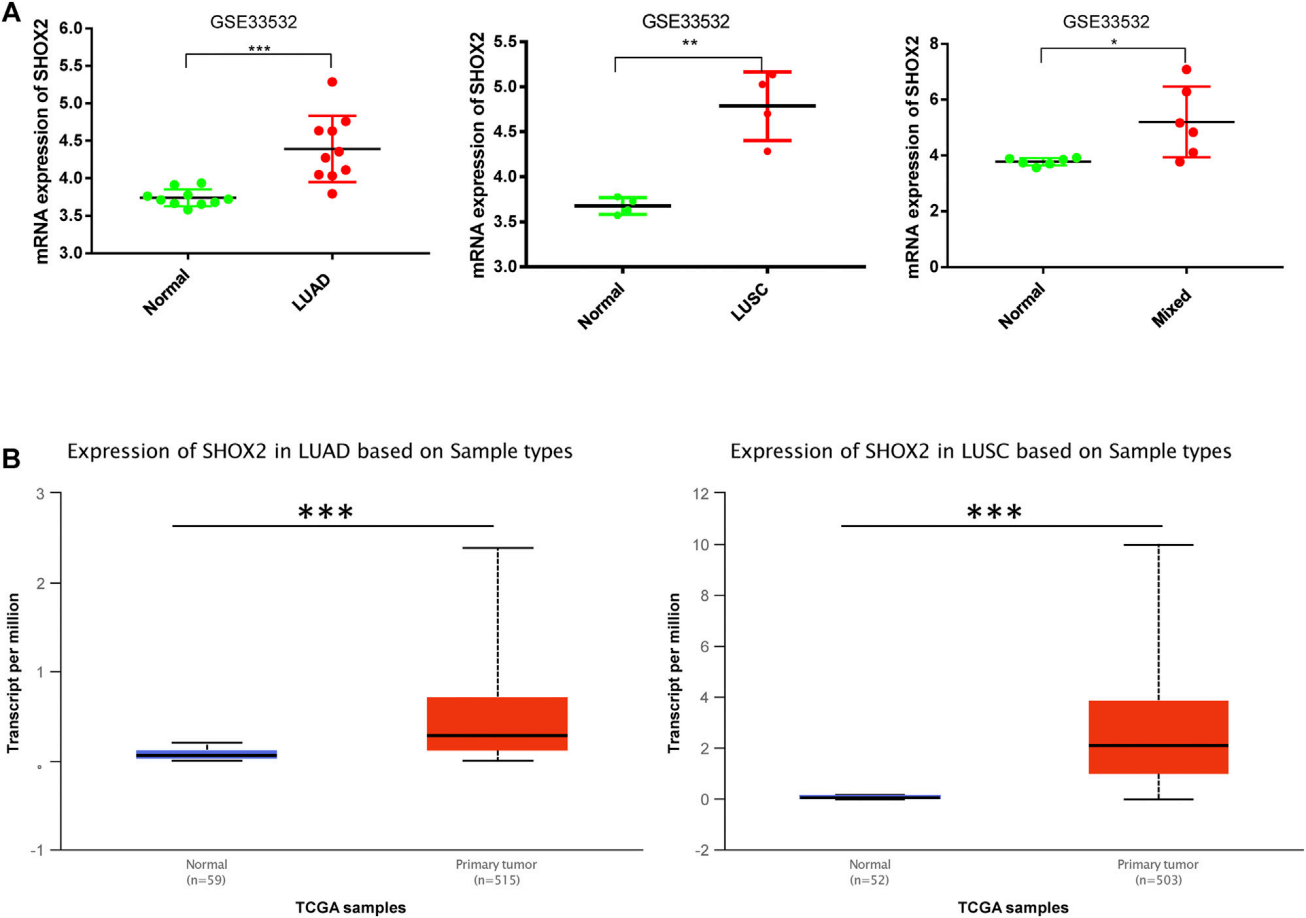
Box plot showing the relative expression of *SHOX2* in normal and NSCLC samples. **(A)** *SHOX2* expression is shown by microarray analysis of 20 NSCLC patients in GSE33532 including 10 LUAD patients, 4 LUSC patients, and 6 mixed patients. These data are compared with those of paired normal tissues. The data are addressed by GraphPad Prism 7.0 and applied paired *t*-test. **(B)** The expression levels of *SHOX2* between normal and LUAD or LUSC tissues are shown in the box plot by UALCAN online analysis tool application. *** represents *p* < 0.001 (highly significant); ** represents *p* < 0.01 (very significant); * represents *p* < 0.05 (significant); LUAD: lung adenocarcinoma; LUSC: lung squamous carcinoma; NSCLC: non-small-cell lung cancer.

In order to explore *SHOX2* mRNA expression and its relationship with clinical features, we addressed the GSE33532 data using GraphPad Prism 7 and used UALCAN to analyze the relative clinical data of LUAD/LUSC patients from TCGA. The *SHOX2* mRNA expression profiling dataset of NSCLC showed that *SHOX2* is highly expressed in both LUAD and LUSC tissues compared to paired normal tissues (Figure 2A). The difference in *SHOX2* expression was statistically significant. This result was verified by an expanded sample size analysis using the online analysis database UALCAN. As shown in Figure 2B, we obtained consistent results.

### The Relationship of *SHOX2* Expression With the Clinical Characteristics of LUAD Patients

**FIGURE 3:**
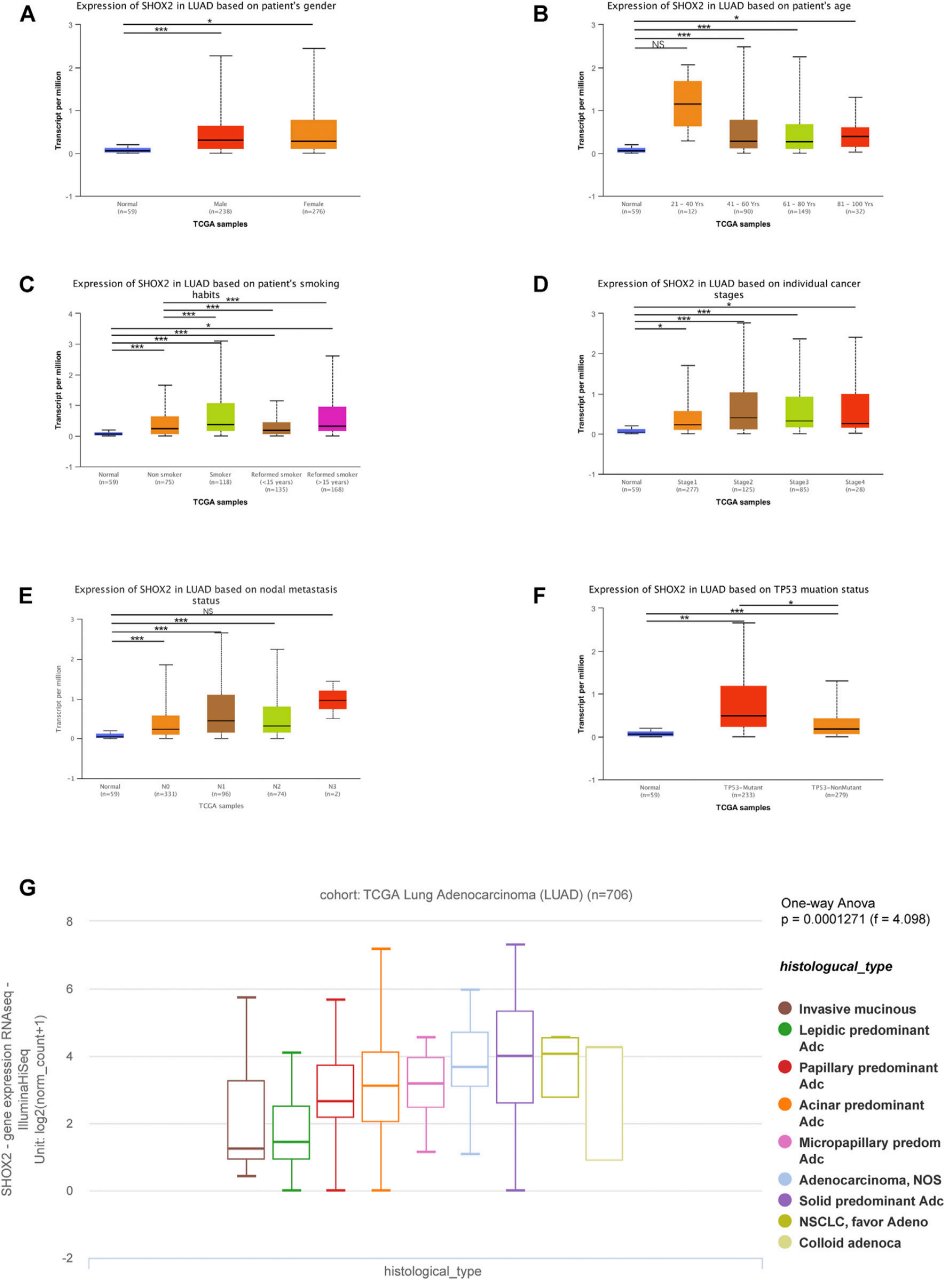
Box plot showing the characteristic relationships of **(A)** gender, **(B)** age, **(C)** smoking habits, **(D)** stage, **(E)** nodal metastasis, **(F)** TP-53 mutation status, and **(G)** histological subtypes with *SHOX2* mRNA expression in LUAD analyzed using the online tools UALCAN and UCSC Xena. Data are represented as mean ± SE. *** represents *p* < 0.001 (highly significant); ** represents *p* < 0.01 (very significant); * represents *p* < 0.05 (significant); LUAD: lung adenocarcinoma.

In recent decades, the incidence of LUAD has been higher than that of LUSC. Moreover, there are few reports on the relationship between *SHOX2* expression and the clinicopathological features of LUAD. Therefore, we used the UALCAN online analysis tool to address *SHOX2* mRNA expression in different subgroups having multiple clinicopathological features from LUAD samples from TCGA. The *SHOX2* mRNA expression level was significantly higher in LUAD patients than in healthy people in subgroup analyses based on sex, age, smoking habits, stage, nodal metastasis, and TP-53 mutation status (Figures 3A–F). In addition, subgroup analyses showed that *SHOX2* expression was higher in patients aged 20–40 years, with a history of smoking, in stage 2, and with nodal distant metastasis, compared to other clinical features (Figure 3). The pathology of disease can help clinicians to clearly diagnose the disease and take reasonable and effective treatment measures. UCSC Xena was used to analyze the changes of *SHOX2* expression in different histological subtypes of invasive LUAD from a cohort of TCGA LUAD (*n* = 706) (Figure 3G). The results showed that *SHOX2* expression varied significantly among different histological subtypes of LUAD (one-way ANOVA, *p* = 0.0001271, *f* = 4.098). *SHOX2* expression was higher in subtypes prone to lymph node metastasis, prone to recurrence, with poor differentiation, and with poor prognosis.

**FIGURE 4:**
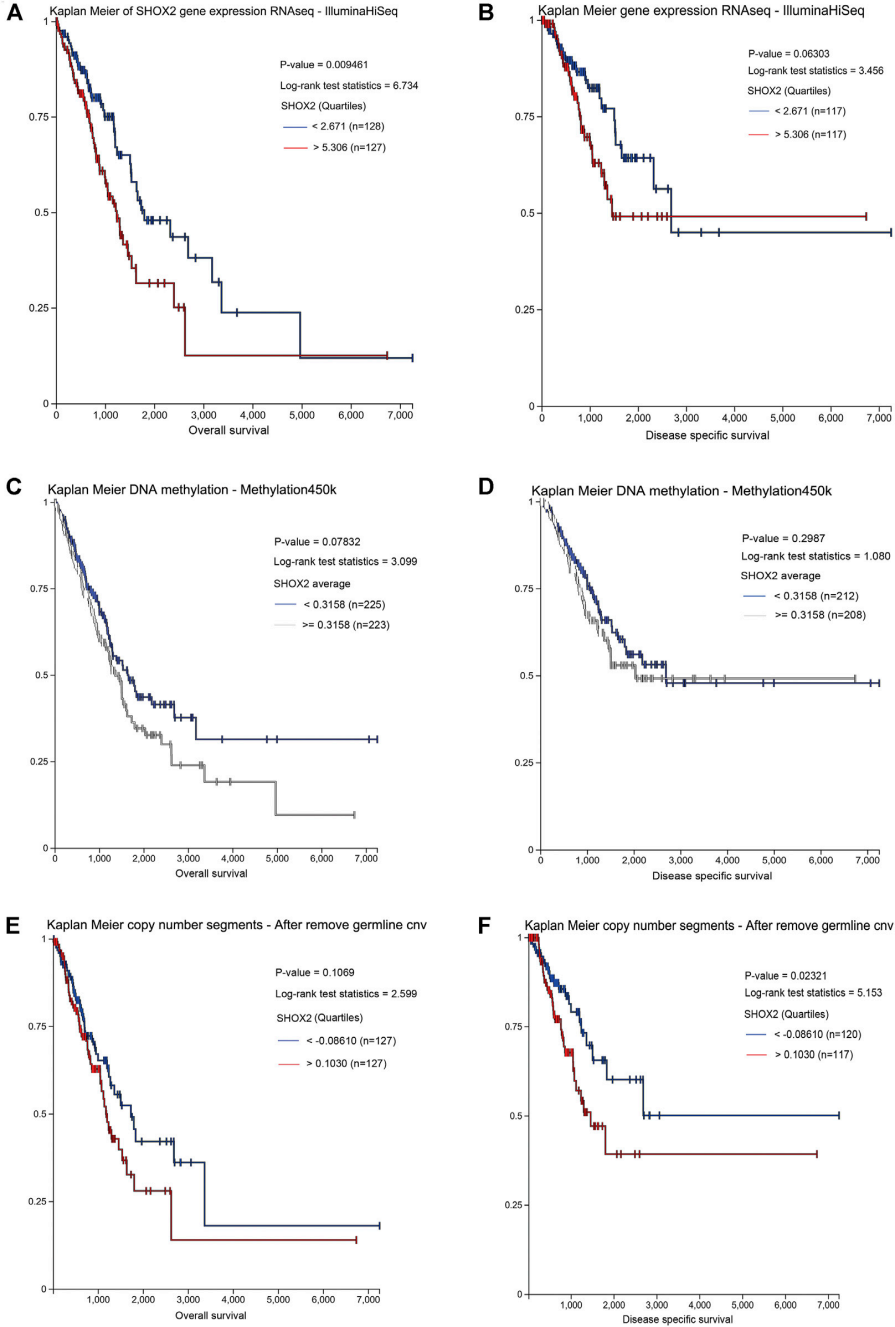
Prognostic value of the *SHOX2* mRNA expression level in LUAD patients by UCSC Xena (Kaplan–Meier plotter). **(A)** Relationship between *SHOX2* expression and OS in LUAD. **(B)** Relationship between *SHOX2* expression and DSS in LUAD. **(C)** Relationship between *SHOX2* DNA methylation and OS in LUAD. **(D)** Relationship between *SHOX2* DNA methylation and DSS in LUAD. **(E)** Relationship between *SHOX2* copy number segments and OS in LUAD. **(F)** Relationship between *SHOX2* copy number segments and DSS in LUAD. *p* < 0.05 indicates statistical significance. LUAD: lung adenocarcinoma; OS: overall survival; DSS: disease-specific survival.

Furthermore, to confirm the prognostic value of SHOX2 in LUAD, UCSC Xena was searched to investigate the effects of *SHOX2* expression, methylation, and CNV on overall survival (OS) and disease-specific survival (DSS). It was confirmed that high *SHOX2* mRNA expression was significantly associated with decreased OS and DSS time in LUAD (*p* < 0.05); DNA methylation and copy number variation had no significant effect on OS (*p* > 0.05), and a higher copy number indicated a shorter DSS (*p* < 0.05). The *SHOX2* expression level and DNA methylation level had no significant correlation with DSS (*p* > 0.05) (Figure 4).

Therefore, these results suggest that *SHOX2* might be a driving factor in the development of LUAD and may act as a potential diagnostic and prognostic indicator in LUAD.

### Influencing-Factor Analysis of *SHOX2* Gene Expression in LUAD

**FIGURE 5:**
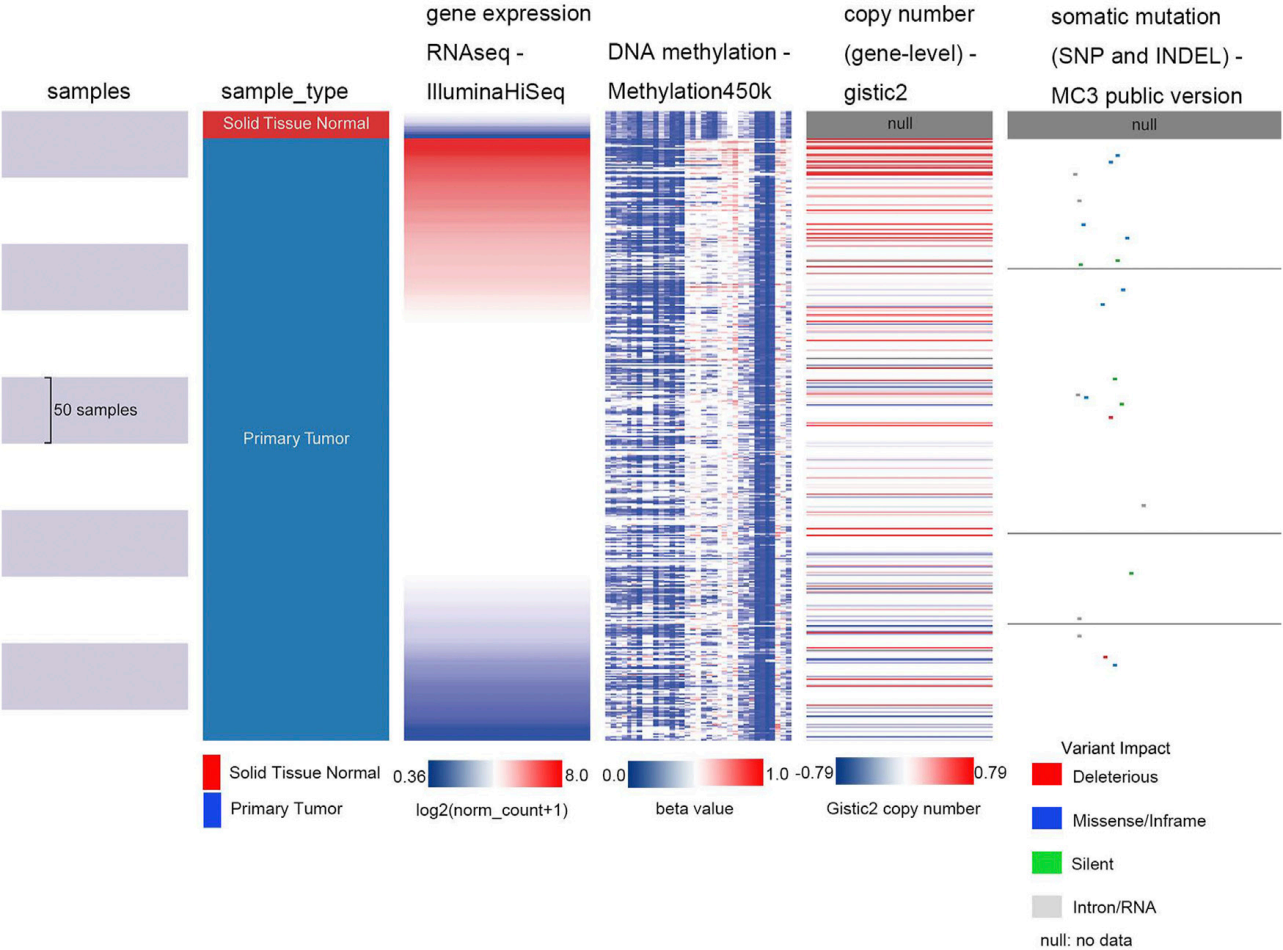
Heat map of *SHOX2* mRNA expression, methylation, copy number, and somatic mutation in patients with primary LUAD and normal tissue. The data are obtained from TCGA-LUAD with a total of 706 samples. LUAD: lung adenocarcinoma.

**FIGURE 6:**
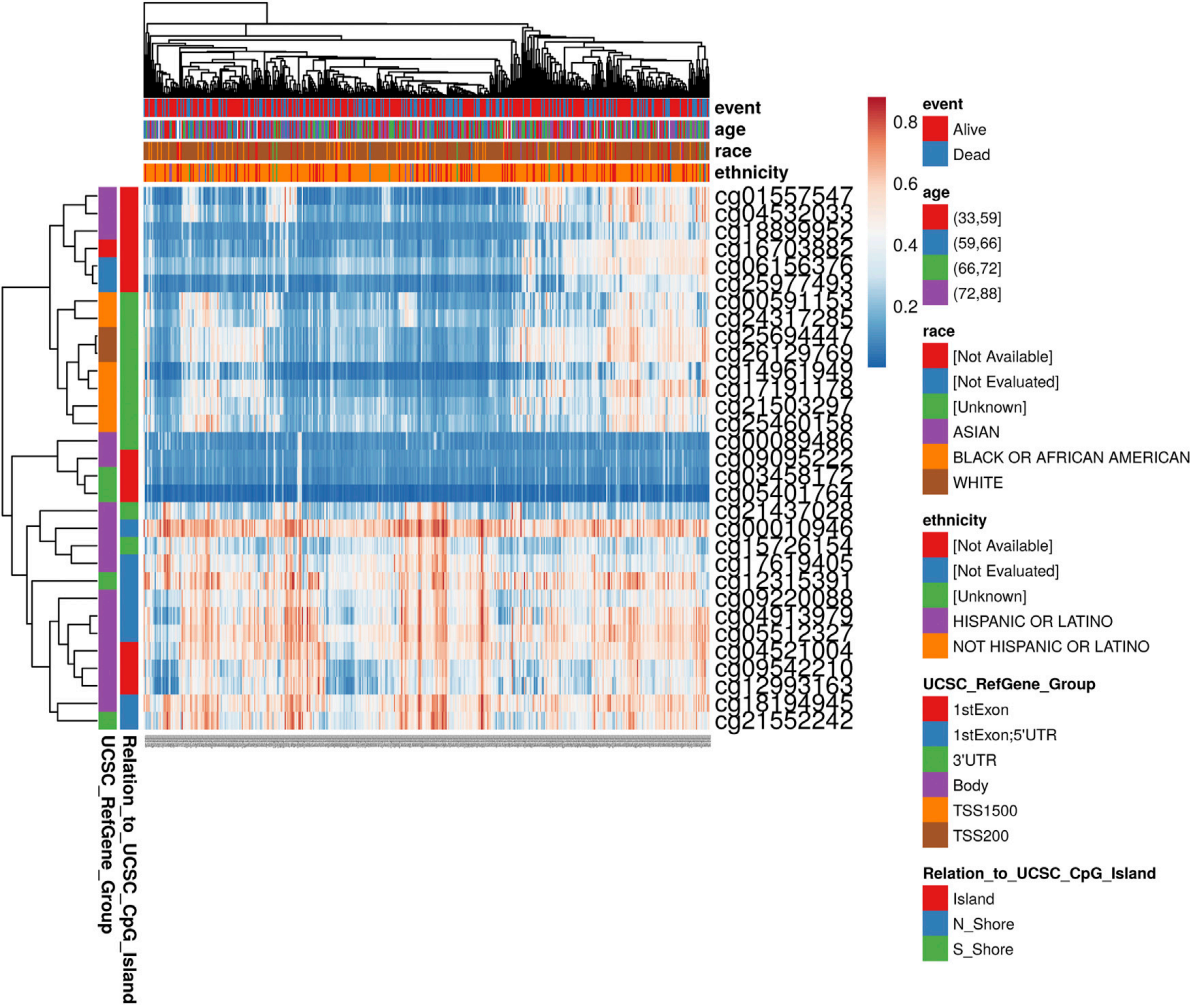
Heat map of methylation levels of the *SHOX2* methylated probe. Red to blue: high expression to low expression. Various colorful side-boxes are used to characterize the event, age, race, ethnicity, UCSC_refGene_Group, and relation to UCSC_CpG_island.

To determine the main influencing factors of *SHOX2* gene expression, we used several online tools performing mutation, methylation, and CNV alteration analysis. We used three methylation analysis tools (UCSC Xena, SurvivalMeth, and MethSurv) to probe the *SHOX2* methylation level in LUAD patients, from different perspectives. The methylation level of *SHOX2* was examined in TCGA LUAD patients, based on age, race, ethnicity, alive or dead events, *SHOX2* mRNA expression, CpG island–related elements, and transcript-related elements. From Figure 5 and Table 1, we can conclude that DNA methylation of *SHOX2* was elevated in LUAD tissues compared with that in normal lung tissues. As shown in Figure 5, *SHOX2* DNA was only locally methylated. To explore the DNA-methylated specific site of *SHOX2* and verify the acquired results, we conducted an in-depth study using TCGA LUAD patient data in SurvivalMeth and MethSurv. In LUAD tissues, *SHOX2* DNA methylation mainly occurred in the gene body, at the hypomethylation level (*p* < 0.05) (Figures 5 and 6, Tables 1 and 2). According to previous research results, hypomethylation of the gene body leads to the high expression of oncogenes, which is opposite to the hypermethylation of the gene promoter for tumor suppressive genes (Yang et al., 2014). Therefore, hypomethylation of the *SHOX2* gene body might be one of the main factors causing mRNA expression. In conclusion, in LUAD tissue, the mRNA expression of *SHOX2* might be correlated with the DNA methylation level, followed by CNV, but not with gene mutation (Figure 5).

**TABLE 1:** Differential methylation levels of the SHOX2 probe between LUAD and normal samples.

Probe ID	Gene location	CpG island regions	Average methylated data value of tumor samples	Average methylated data value of normal samples	Delta value	Fold change	*p*-Value
cg00591153	TSS1500	S_Shore	0.268614	0.180493	0.088121	1.488224	4.45580E−17
cg14961949	TSS1500	S_Shore	0.173536	0.065364	0.108171	2.654909	3.30727E−35
cg15548431	TSS1500	S_Shore	0.232887	0.171044	0.061843	1.36156	4.02456E−10
cg17191178	TSS1500	S_Shore	0.298609	0.18082	0.117789	1.651417	1.09924E−29
cg21503297	TSS1500	S_Shore	0.300121	0.227304	0.072816	1.320346	1.50735E−15
cg24317285	TSS1500	S_Shore	0.302047	0.237082	0.064965	1.274019	1.20478E−14
cg25460158	TSS1500	S_Shore	0.315503	0.266667	0.048836	1.183137	4.88871E−07
cg25694447	TSS200	S_Shore	0.31945	0.238284	0.081165	1.340622	4.16236E−16
cg26129769	TSS200	S_Shore	0.33075	0.241758	0.088992	1.368102	4.80355E−16
cg16703882	1st EXON	Island	0.249193	0.219976	0.029217	1.13282	4.32357E−03
cg00010946	BODY	N_Shore	0.616993	0.549898	0.067095	1.122014	9.46071E−08
cg00089486	BODY	S_Shore	0.116562	0.096583	0.019978	1.20685	7.74109E−05
cg01557547	BODY	Island	0.241769	0.093843	0.147926	2.576302	6.99470E−43
cg04521004	BODY	Island	0.512873	0.243548	0.269325	2.105839	3.27178E−51
cg04532033	BODY	Island	0.28867	0.137343	0.151326	2.101811	2.20417E−49
cg04913979	BODY	N_Shore	0.479174	0.188612	0.290561	2.540521	5.56975E−49
cg05512327	BODY	N_Shore	0.547018	0.372874	0.174143	1.467029	2.66119E−28
cg09095222	BODY	Island	0.1328	0.122822	0.009977	1.081235	3.59488E−02
cg09220088	BODY	N_Shore	0.424979	0.295727	0.129251	1.437061	4.68587E−21
cg09542210	BODY	Island	0.390491	0.135601	0.254889	2.879692	5.24557E−77
cg12993163	BODY	Island	0.389816	0.103129	0.286687	3.779884	9.59716E−121
cg15726154	BODY	S_Shore	0.350172	0.307982	0.04219	1.136988	2.95931E−03
cg18194945	BODY	N_Shore	0.542494	0.300898	0.241596	1.802915	2.95084E−28
cg18899952	BODY	Island	0.173432	0.107374	0.066058	1.615213	1.51266E−19
cg20501518	BODY	N_Shore	0.401894	0.183032	0.218862	2.19576	7.34036E−40
cg21437028	BODY	S_Shore	0.317038	0.267349	0.049689	1.185859	3.47849E−04
cg21847036	BODY	S_Shore	0.35551	0.304502	0.051008	1.167512	5.67448E−04
cg25506747	3′UTR	N_Shore	0.33401	0.169969	0.164041	1.965125	2.02697E−25
cg21552242	3′UTR	N_Shore	0.479399	0.276377	0.203021	1.734581	2.60443E−33
cg12315391	3′UTR	N_Shore	0.537819	0.425058	0.112761	1.265283	6.56708E−14
cg05401764	3′UTR	Island	0.045138	0.037674	0.007464	1.198133	3.45000E−02

Note: DNA methylation level is represented by data value (0 represents unmethylated, 0.25–0.3 represents hypomethylated, 0.5–0.7 represents hypermethylated, and 1 represents completely methylated).

**TABLE 2:** Average data value of the SHOX2 methylated probe between LUAD and normal samples.

Gene location	Average data value of the methylated probe in tumor samples	Average data value of the methylated probe in normal samples
Promoter	0.282390778	0.200979556
1st exon	0.249193	0.219976
Body	0.369510882	0.224183353
3′UTR	0.3490915	0.2272695

Note: DNA methylation level is represented by data value (0 represents unmethylated, 0.25–0.3 represents hypomethylated, 0.5–0.7 represents hypermethylated, and 1 represents completely methylated).

### Enrichment Analysis of Correlated Genes With *SHOX2* in LUAD

**FIGURE 7:**
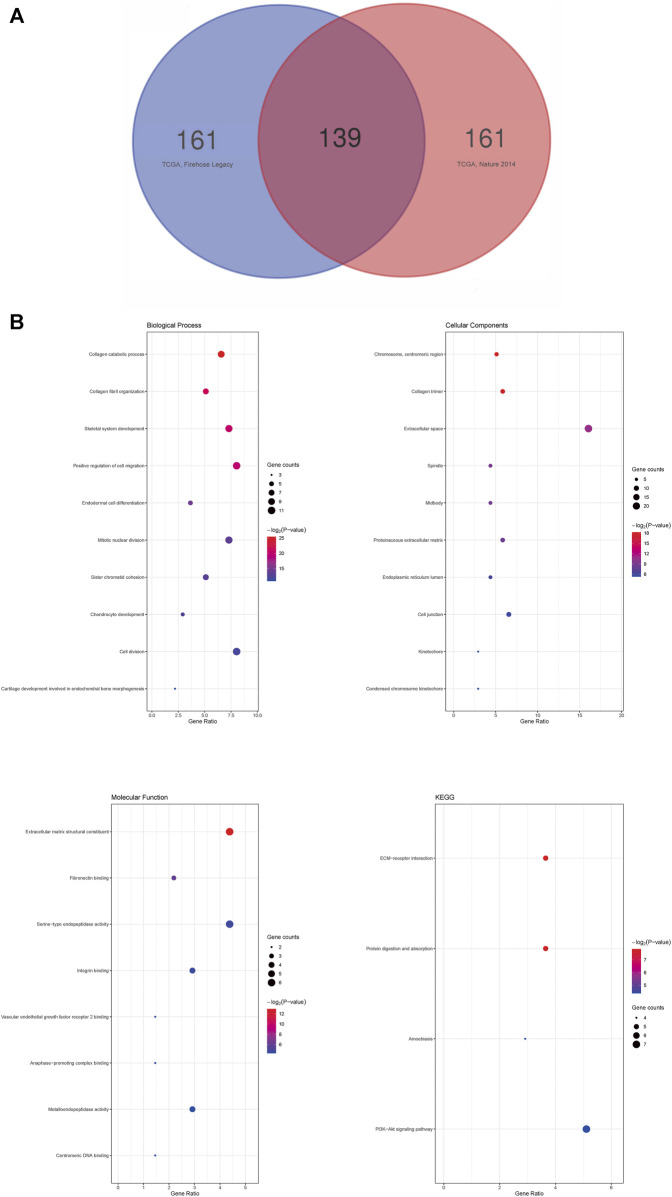
Enrichment analysis of correlated genes with *SHOX2* in LUAD. **(A)** Venn diagram of *SHOX2* correlated genes in LUAD. The 139 significantly correlated genes with *SHOX2* appeared in two TCGA research cohorts, Firehose Legacy (586 samples) and Nature 2014 (230 samples). **(B)** GO and KEGG analyses of *SHOX2* and the significantly correlated genes by DAVID database. The node size represents the gene ratio; the node color represents the *p*-value. LUAD: lung adenocarcinoma; GO: Gene Ontology; KEGG: Kyoto Encyclopedia of Genes and Genomes.

Finally, to explore the possible signaling pathways from the genes correlated with *SHOX2*, we analyzed mRNA sequencing data from 816 LUAD patients in two TCGA research cohorts: Firehose Legacy (586 samples) and Nature 2014 (230 samples). As shown in the Venn diagram (Figure 7A), 139 significantly correlated genes with *SHOX2* appeared in both cohorts (*p* < 0.05). This suggests that *SHOX2* has a widespread impact on the transcriptome. We then performed functional enrichment analysis of *SHOX2* and these 139 common significantly correlated genes using the DAVID database. The top 10 GO terms of these genes, according to the gene counts, are shown in Figure 7B, including biological processes (BPs), cellular components (CCs), and molecular functions (MFs). The BP of *SHOX2* was mainly associated with positive regulation of cell migration, cell division, skeletal system development, and mitotic nuclear division. The CC of *SHOX2* was mainly located in the extracellular space. The MF of *SHOX2* mainly focuses on the structural constituents of the extracellular matrix and serine-type endopeptidase activity. Surprisingly, the significant KEGG pathways included the PI3K–Akt signaling pathway, ECM–receptor interaction, protein digestion and absorption, and amebiasis (*p* < 0.05).

## Discussion

Early detection, early diagnosis, and early treatment are of key importance in preventing disease development. At present, conventional diagnostic methods for lung cancer include computed tomography imaging and cellular/histopathological examination. Emerging molecular diagnostic methods are being increasingly applied in clinical practice because they are more sensitive and objective. DNA methylation alteration is considered an early predictor of cancer and can be detected during the early stages of tumorigenesis (Zhang Y. et al., 2016; Dirks et al., 2016; Wang et al., 2018; Srisuttee et al., 2020). Because of the hypermethylation of *SHOX2* in lung cancer, methylation detection has been applied to assist in the early diagnosis of unknown pulmonary nodules (Kneip et al., 2011; Konecny et al., 2016; Weiss et al., 2017; Shi et al., 2020). Many studies have reported that *SHOX2* is highly expressed in many cancers. The specific role of *SHOX2* in lung cancer patients and the molecular mechanism of its action are unknown.

As reported in detail in our previous review (Li et al., 2020), we synthesized the results of existing studies on *SHOX2* and its related homonymous genes in mice, lung cancer and other tumors and concluded that *SHOX2* might play an important role in tumorigenesis, metastasis, and recurrence in lung cancer. Here, we used bioinformatics analysis to verify the above conclusions.

In our study, we found that the mRNA expression of *SHOX2* was higher in multiple cancers than in paired normal tissues, including LUAD and LUSC tissues. Among the LUAD patients, *SHOX2* expression was higher in patients of middle–young age, with smoking history, in advanced stages, and with nodal distant metastasis. In addition, our results showed that patients with high expression of *SHOX2* are prone to recurrence, poor differentiation, and poor prognosis. Here, we identified that *SHOX2* plays a negative role in LUAD progression and could act as an oncogene. Next, we performed *SHOX2* methylation analysis. Finally, we found that *SHOX2* undergoes hypomethylation in the gene body. The main factors for *SHOX2* mRNA expression are the DNA methylation and CNV, but not gene mutations in LUAD. Additionally, *SHOX2* has cross talk in the PI3K−Akt signaling pathway and in ECM–receptor interactions.

The potential role of *SHOX2* in patients with lung cancer may have been explained by many previous studies. We identified that the mRNA expression of *SHOX2* could assist in indicating subsets of LUAD with worse prognosis and survival. Zhang and Zhou also reported that *SHOX2* was an indicator for identifying subgroups with worse prognosis in lower-grade gliomas, which is consistent with our result (Zhang Y.A. et al., 2016). Additionally, there have been other similar reports of *SHOX2* methylation as a predictor of malignancy (Leiro et al., 2019; Xu et al., 2020).

Tumor cell genomes showed global hypomethylation and local hypermethylation. Local hypermethylation may occur on the promoter CpG island of a tumor suppressor gene to inhibit the expression of this gene, or on the gene body CpG island of an oncogene to induce high expression of this gene (Yang et al., 2014; Gagliardi et al., 2018). Downregulated TSGs or upregulated oncogenes are critical in tumorigenesis. According to recent research, *SHOX2* is considered an oncogene. Unexpectedly, the present majority of related reports declare that *SHOX2* methylation occurs on the promoter CpG island, which can seem incomprehensible. To explain the discrepancy of high methylation of *SHOX2* in cancer tissue and to identify whether methylation occurs at the gene promoter, we conducted further research to try to explain this phenomenon. We used UCSC XENA, SurvivalMeth, and MethSurv to obtain methylation data for all methylation sites of *SHOX2* and then analyzed them statistically using Excel. Interestingly, we observed that the *SHOX2* methylation is in a hypomethylated state, and methylation mainly occurs at the gene body instead of at the gene promoter. In summary, *SHOX2* is highly expressed, and its genomes are hypomethylated in LUAD, which might be the main mechanism of gene expression. Therefore, the current description of *SHOX2* promoter hypermethylation may not be rigid.

The regulation of gene expression is the molecular basis of cell differentiation, morphogenesis, and ontogenesis *in vivo*. The influencing factors of gene expression include one or two of the following alterations in most cases: local mutation, CNV, DNA methylation, and the expression level of master transcription factors on another chromosome. We found that hypomethylation of the *SHOX2* gene body may be one of the main mechanisms driving the upregulation of *SHOX2*, and CNV is also one of them. Schneider et al. (2011) and Zhang Y.-A. et al. (2016) may agree with our findings.

According to our enrichment analysis of *SHOX2*, the BP of *SHOX2* was mainly associated with positive regulation of cell migration, cell division, skeletal system development, and mitotic nuclear division, and the significant KEGG pathways included the PI3K−Akt signaling pathway, ECM−receptor interaction, protein digestion and absorption, and amebiasis. These findings are consistent with the fact that *SHOX2* is considered an oncogene and a diagnostic and predicted biomarker. This is critical for understanding how *SHOX2* expression alter and how *SHOX2* lead to cancers such as LUAD.

In this study, we combined multiple online bioinformatics data analysis platforms and tools to provide a statistical analysis of the correlation between *SHOX2* expression and clinical characteristic parameters, clinical prognosis, DNA methylation, CNV, mutations, co-expressed genes, and related signaling pathways in LUAD. *SHOX2* is highly expressed in most cancers. *SHOX2* gene expression may be mainly regulated by methylation of the gene body in LUAD, and its high expression or hypomethylation indicates poor differentiation and poor prognosis. *SHOX2* is involved in PI3K−Akt and other important cancer-related signaling pathways to promote tumorigenesis.

## Data Availability

The datasets presented in this study can be found in online repositories. The names of the repository/repositories and accession number(s) can be found in the article/Supplementary Material.
